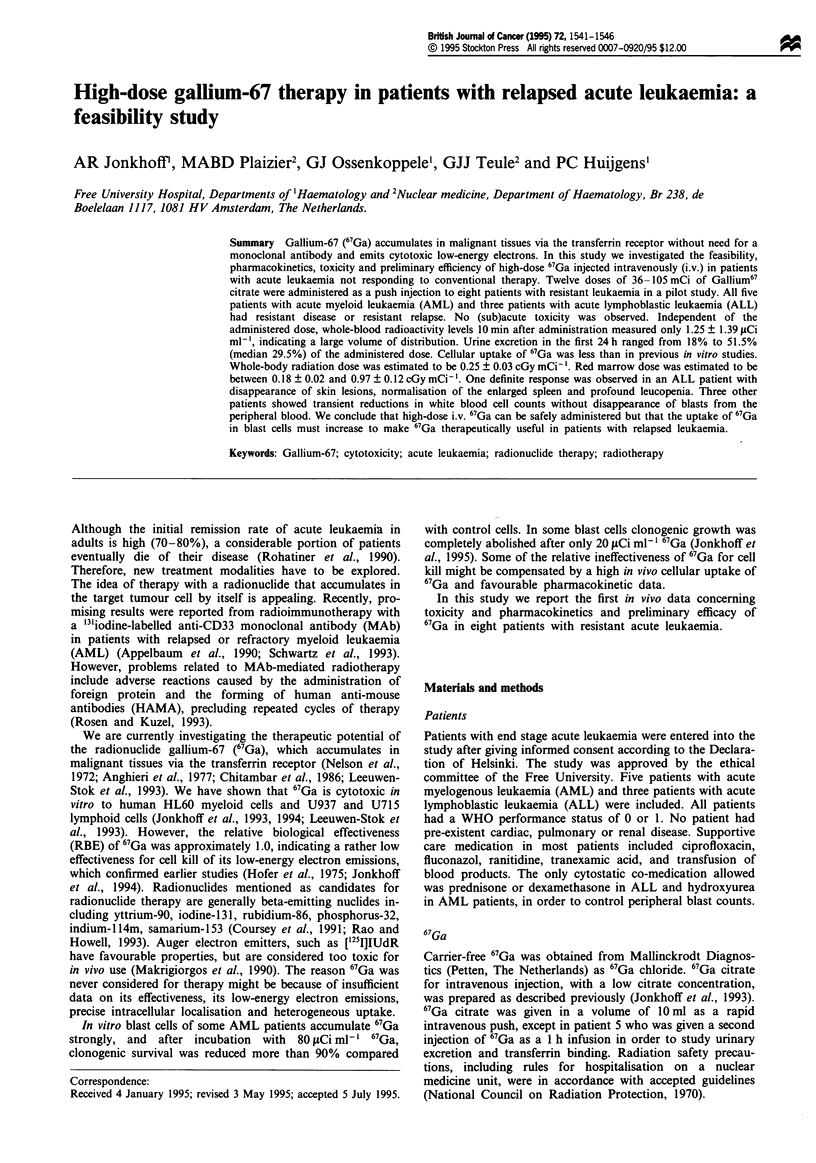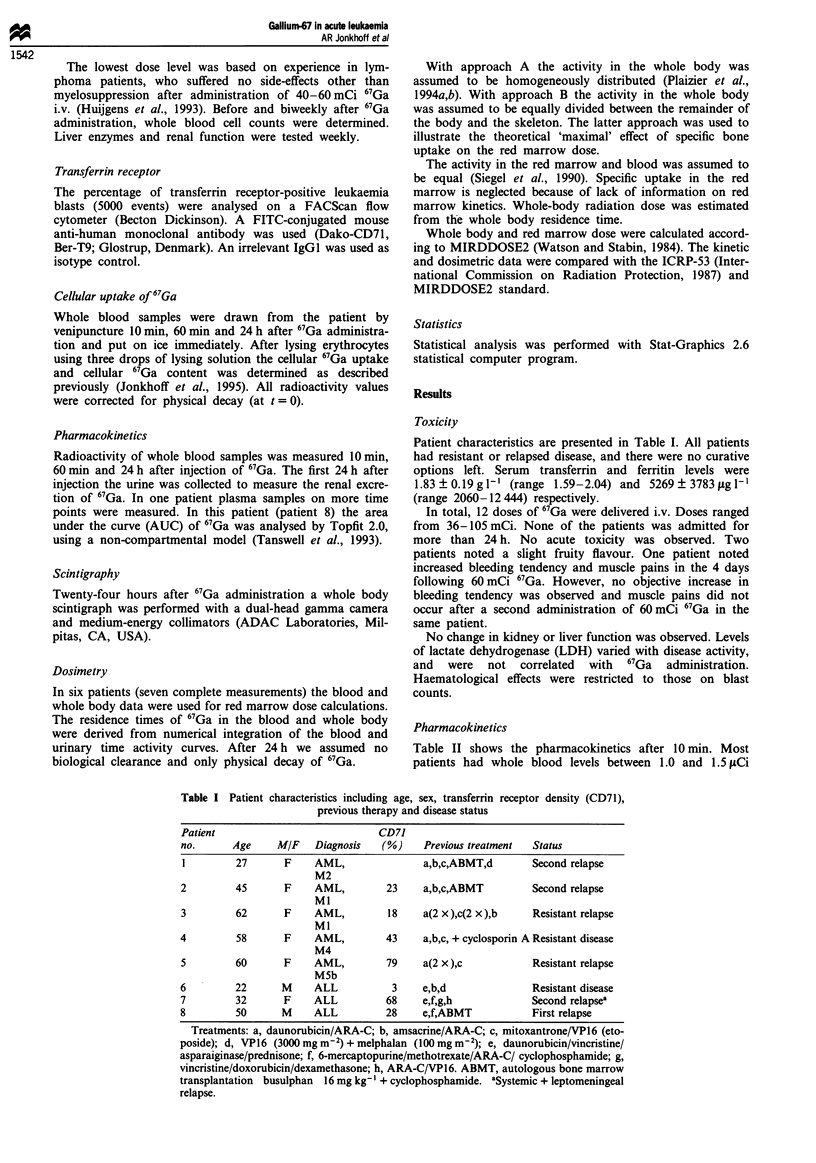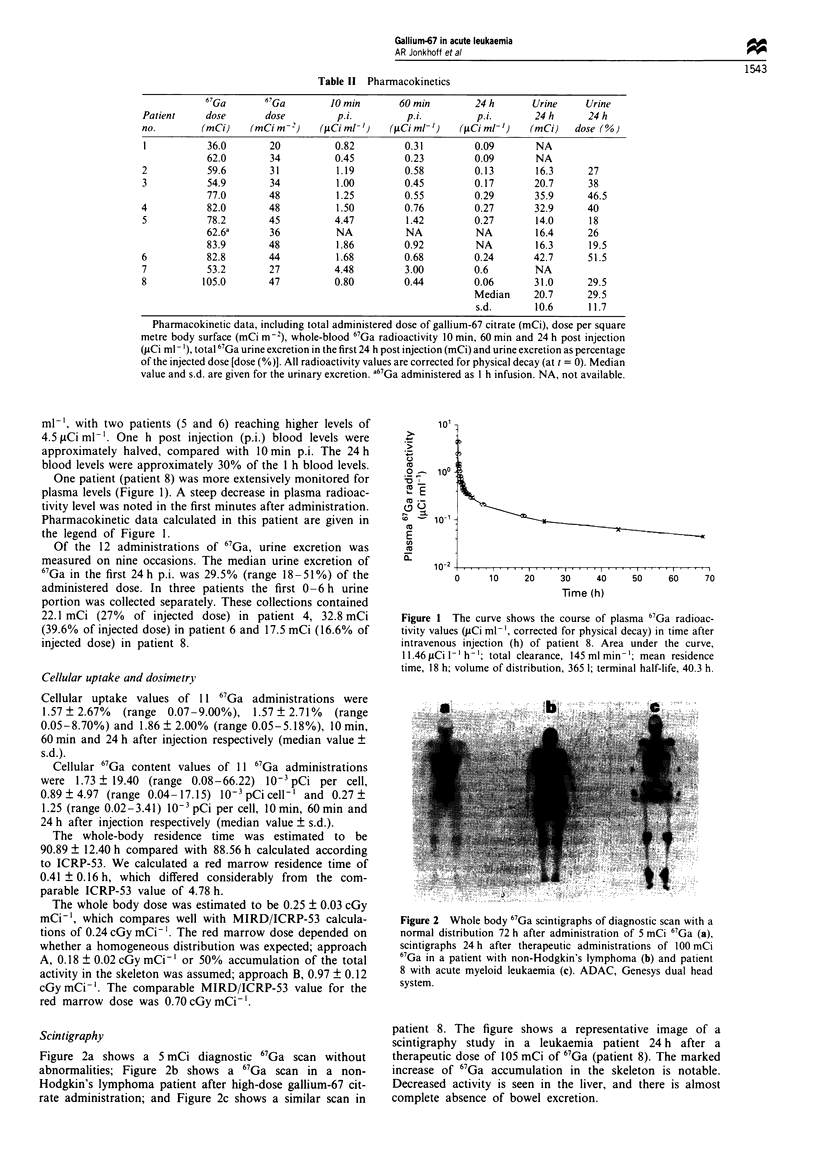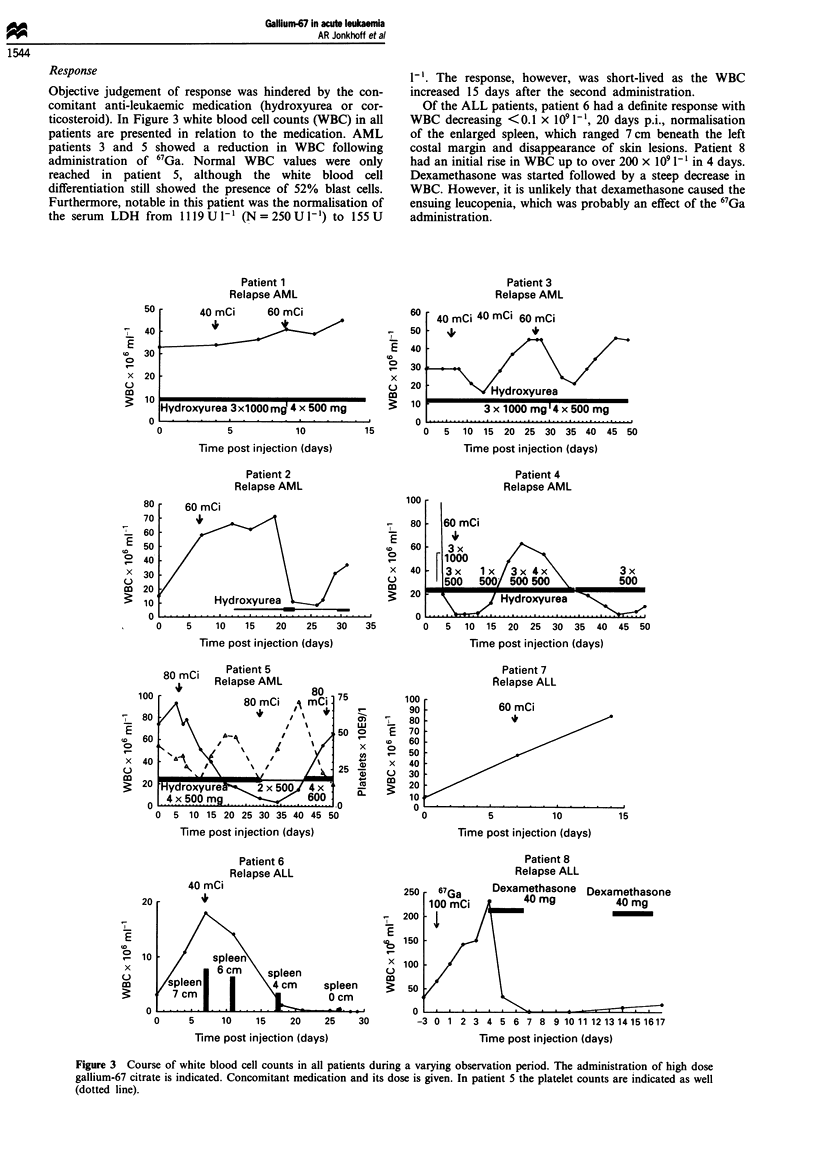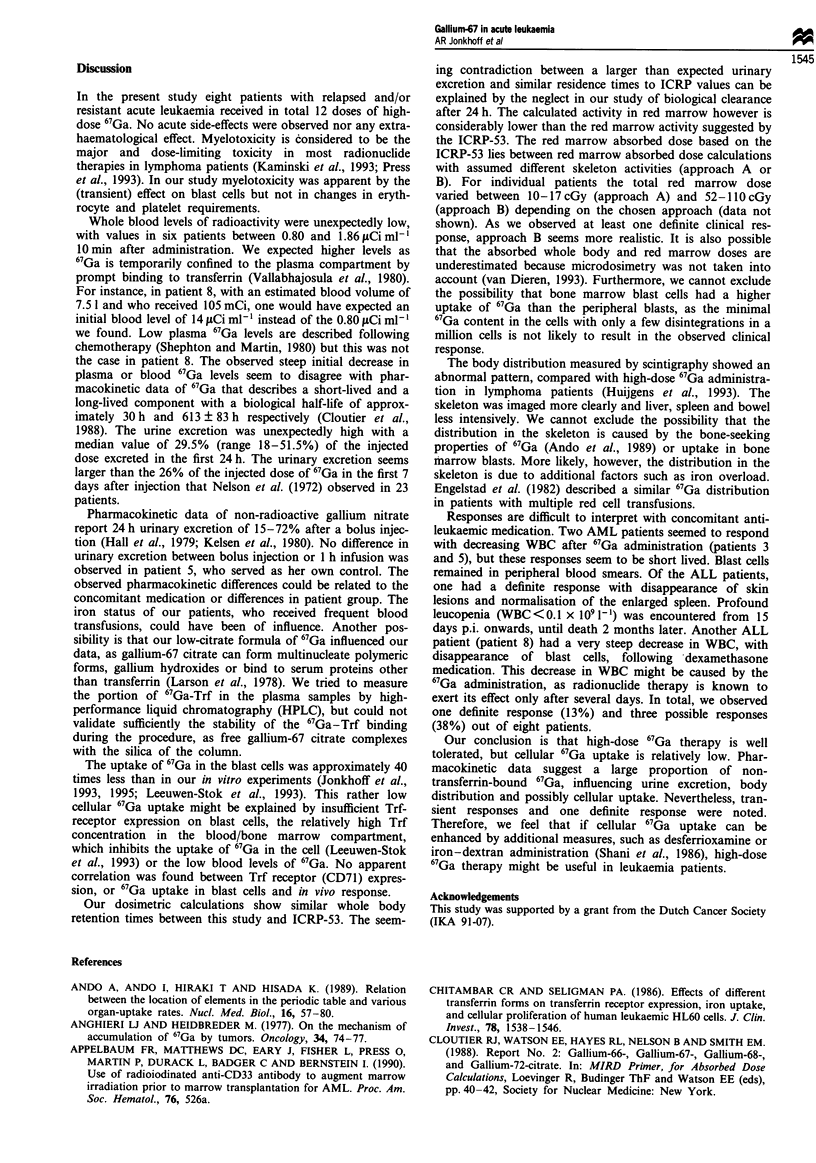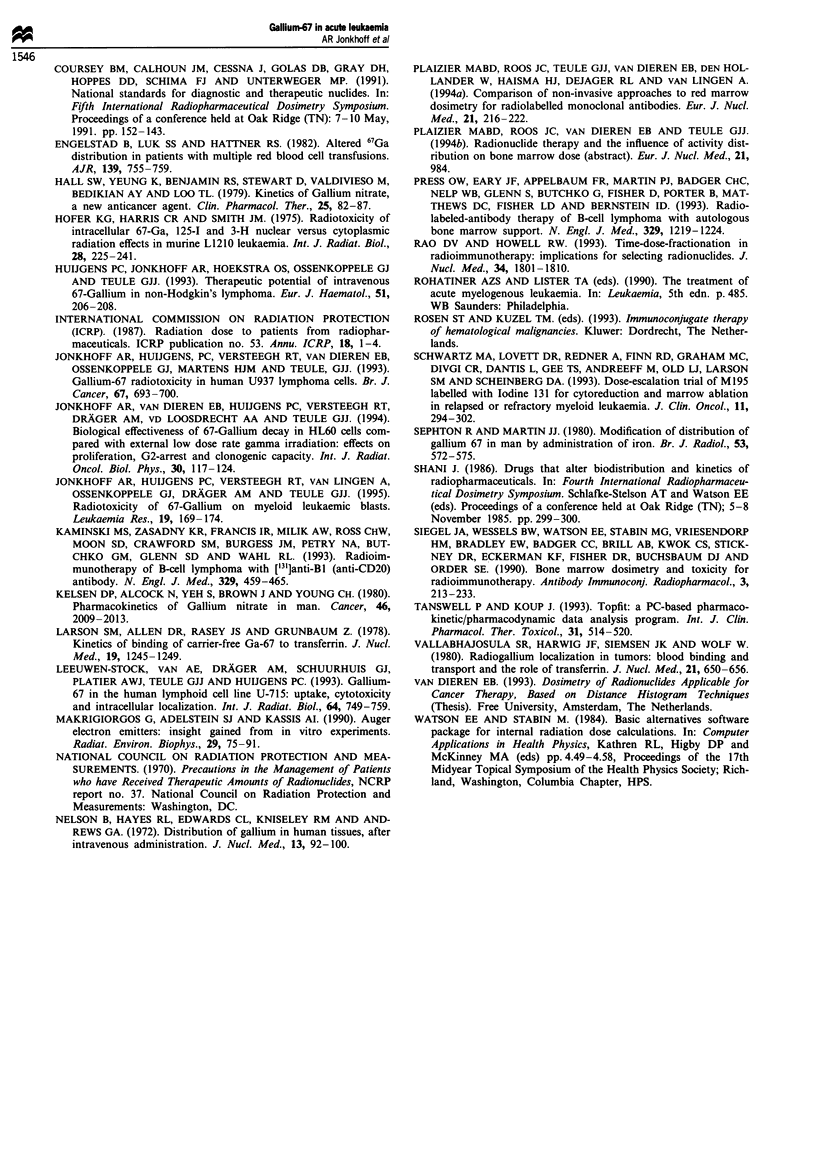# High-dose gallium-67 therapy in patients with relapsed acute leukaemia: a feasibility study.

**DOI:** 10.1038/bjc.1995.544

**Published:** 1995-12

**Authors:** A. R. Jonkhoff, M. A. Plaizier, G. J. Ossenkoppele, G. J. Teule, P. C. Huijgens

**Affiliations:** Free University Hospital, Department of Haematology, Amsterdam, The Netherlands.

## Abstract

**Images:**


British Journal of Cancer (1995) 72, 1541–1546
© 1995 Stockton Press All rights reserved 0007-0920/95 $12.00

### High-dose gallium-67 therapy in patients with relapsed acute leukaemia: a feasibility study

AR Jonkhoff[1], MABD Plaizier[2], GJ Ossenkoppele[1], GJJ Teule[2] and PC Huijgens[1]

*Free University Hospital, Departments of [1]Haematology and [2]Nuclear medicine, Department of Haematology, Br 238, de Boelelaan 1117, 1081 HV Amsterdam, The Netherlands.*

**Summary** Gallium-67 ($^{67}$Ga) accumulates in malignant tissues via the transferrin receptor without need for a monoclonal antibody and emits cytotoxic low-energy electrons. In this study we investigated the feasibility, pharmacokinetics, toxicity and preliminary efficiency of high-dose $^{67}$Ga injected intravenously (i.v.) in patients with acute leukaemia not responding to conventional therapy. Twelve doses of 36–105 mCi of Gallium$^{67}$ citrate were administered as a push injection to eight patients with resistant leukaemia in a pilot study. All five patients with acute myeloid leukaemia (AML) and three patients with acute lymphoblastic leukaemia (ALL) had resistant disease or resistant relapse. No (sub)acute toxicity was observed. Independent of the administered dose, whole-blood radioactivity levels 10 min after administration measured only 1.25 ± 1.39 $\mu$Ci ml$^{-1}$, indicating a large volume of distribution. Urine excretion in the first 24 h ranged from 18% to 51.5% (median 29.5%) of the administered dose. Cellular uptake of $^{67}$Ga was less than in previous *in vitro* studies. Whole-body radiation dose was estimated to be 0.25 ± 0.03 cGy mCi$^{-1}$. Red marrow dose was estimated to be between 0.18 ± 0.02 and 0.97 ± 0.12 cGy mCi$^{-1}$. One definite response was observed in an ALL patient with disappearance of skin lesions, normalisation of the enlarged spleen and profound leucopenia. Three other patients showed transient reductions in white blood cell counts without disappearance of blasts from the peripheral blood. We conclude that high-dose i.v. $^{67}$Ga can be safely administered but that the uptake of $^{67}$Ga in blast cells must increase to make $^{67}$Ga therapeutically useful in patients with relapsed leukaemia.

**Keywords**: Gallium-67; cytotoxicity; acute leukaemia; radionuclide therapy; radiotherapy

Although the initial remission rate of acute leukaemia in adults is high (70–80%), a considerable portion of patients eventually die of their disease (Rohatiner *et al.*, 1990). Therefore, new treatment modalities have to be explored. The idea of therapy with a radionuclide that accumulates in the target tumour cell by itself is appealing. Recently, promising results were reported from radioimmunotherapy with a $^{131}$iodine-labelled anti-CD33 monoclonal antibody (MAb) in patients with relapsed or refractory myeloid leukaemia (AML) (Appelbaum *et al.*, 1990; Schwartz *et al.*, 1993). However, problems related to MAb-mediated radiotherapy include adverse reactions caused by the administration of foreign protein and the forming of human anti-mouse antibodies (HAMA), precluding repeated cycles of therapy (Rosen and Kuzel, 1993).

We are currently investigating the therapeutic potential of the radionuclide gallium-67 ($^{67}$Ga), which accumulates in malignant tissues via the transferrin receptor (Nelson *et al.*, 1972; Anghieri *et al.*, 1977; Chitambar *et al.*, 1986; Leeuwen-Stok *et al.*, 1993). We have shown that $^{67}$Ga is cytotoxic *in vitro* to human HL60 myeloid cells and U937 and U715 lymphoid cells (Jonkhoff *et al.*, 1993, 1994; Leeuwen-Stok *et al.*, 1993). However, the relative biological effectiveness (RBE) of $^{67}$Ga was approximately 1.0, indicating a rather low effectiveness for cell kill of its low-energy electron emissions, which confirmed earlier studies (Hofer *et al.*, 1975; Jonkhoff *et al.*, 1994). Radionuclides mentioned as candidates for radionuclide therapy are generally beta-emitting nuclides including yttrium-90, iodine-131, rubidium-86, phosphorus-32, indium-114m, samarium-153 (Coursey *et al.*, 1991; Rao and Howell, 1993). Auger electron emitters, such as [$^{125}$I]IUdR have favourable properties, but are considered too toxic for *in vivo* use (Makrigiorgos *et al.*, 1990). The reason $^{67}$Ga was never considered for therapy might be because of insufficient data on its effectiveness, its low-energy electron emissions, precise intracellular localisation and heterogeneous uptake.

*In vitro* blast cells of some AML patients accumulate $^{67}$Ga strongly, and after incubation with 80 $\mu$Ci ml$^{-1}$ $^{67}$Ga, clonogenic survival was reduced more than 90% compared with control cells. In some blast cells clonogenic growth was completely abolished after only 20 $\mu$Ci ml$^{-1}$ $^{67}$Ga (Jonkhoff *et al.*, 1995). Some of the relative ineffectiveness of $^{67}$Ga for cell kill might be compensated by a high *in vivo* cellular uptake of $^{67}$Ga and favourable pharmacokinetic data.

In this study we report the first *in vivo* data concerning toxicity and pharmacokinetics and preliminary efficacy of $^{67}$Ga in eight patients with resistant acute leukaemia.

Correspondence:
Received 4 January 1995; revised 3 May 1995; accepted 5 July 1995.

### Materials and methods

### *Patients*

Patients with end stage acute leukaemia were entered into the study after giving informed consent according to the Declaration of Helsinki. The study was approved by the ethical committee of the Free University. Five patients with acute myelogenous leukaemia (AML) and three patients with acute lymphoblastic leukaemia (ALL) were included. All patients had a WHO performance status of 0 or 1. No patient had pre-existent cardiac, pulmonary or renal disease. Supportive care medication in most patients included ciprofloxacin, fluconazol, ranitidine, tranexamic acid, and transfusion of blood products. The only cytostatic co-medication allowed was prednisone or dexamethasone in ALL and hydroxyurea in AML patients, in order to control peripheral blast counts.

### *$^{67}$Ga*

Carrier-free $^{67}$Ga was obtained from Mallinckrodt Diagnostics (Petten, The Netherlands) as $^{67}$Ga chloride. $^{67}$Ga citrate for intravenous injection, with a low citrate concentration, was prepared as described previously (Jonkhoff *et al.*, 1993). $^{67}$Ga citrate was given in a volume of 10 ml as a rapid intravenous push, except in patient 5 who was given a second injection of $^{67}$Ga as a 1 h infusion in order to study urinary excretion and transferrin binding. Radiation safety precautions, including rules for hospitalisation on a nuclear medicine unit, were in accordance with accepted guidelines (National Council on Radiation Protection, 1970).

The lowest dose level was based on experience in lymphoma patients, who suffered no side-effects other than myelosuppression after administration of 40–60 mCi $^{67}$Ga i.v. (Huijgens *et al.*, 1993). Before and biweekly after $^{67}$Ga administration, whole blood cell counts were determined. Liver enzymes and renal function were tested weekly.

*Transferrin receptor*

The percentage of transferrin receptor-positive leukaemia blasts (5000 events) were analysed on a FACScan flow cytometer (Becton Dickinson). A FITC-conjugated mouse anti-human monoclonal antibody was used (Dako-CD71, Ber-T9; Glostrup, Denmark). An irrelevant IgG1 was used as isotype control.

*Cellular uptake of $^{67}$Ga*

Whole blood samples were drawn from the patient by venipuncture 10 min, 60 min and 24 h after $^{67}$Ga administration and put on ice immediately. After lysing erythrocytes using three drops of lysing solution the cellular $^{67}$Ga uptake and cellular $^{67}$Ga content was determined as described previously (Jonkhoff *et al.*, 1995). All radioactivity values were corrected for physical decay (at $t = 0$).

*Pharmacokinetics*

Radioactivity of whole blood samples was measured 10 min, 60 min and 24 h after injection of $^{67}$Ga. The first 24 h after injection the urine was collected to measure the renal excretion of $^{67}$Ga. In one patient plasma samples on more time points were measured. In this patient (patient 8) the area under the curve (AUC) of $^{67}$Ga was analysed by Topfit 2.0, using a non-compartmental model (Tanswell *et al.*, 1993).

*Scintigraphy*

Twenty-four hours after $^{67}$Ga administration a whole body scintigraph was performed with a dual-head gamma camera and medium-energy collimators (ADAC Laboratories, Milpitas, CA, USA).

*Dosimetry*

In six patients (seven complete measurements) the blood and whole body data were used for red marrow dose calculations. The residence times of $^{67}$Ga in the blood and whole body were derived from numerical integration of the blood and urinary time activity curves. After 24 h we assumed no biological clearance and only physical decay of $^{67}$Ga.

With approach A the activity in the whole body was assumed to be homogeneously distributed (Plaizier *et al.*, 1994*a*,*b*). With approach B the activity in the whole body was assumed to be equally divided between the remainder of the body and the skeleton. The latter approach was used to illustrate the theoretical 'maximal' effect of specific bone uptake on the red marrow dose.

The activity in the red marrow and blood was assumed to be equal (Siegel *et al.*, 1990). Specific uptake in the red marrow is neglected because of lack of information on red marrow kinetics. Whole-body radiation dose was estimated from the whole body residence time.

Whole body and red marrow dose were calculated according to MIRDDOSE2 (Watson and Stabin, 1984). The kinetic and dosimetric data were compared with the ICRP-53 (International Commission on Radiation Protection, 1987) and MIRDDOSE2 standard.

*Statistics*

Statistical analysis was performed with Stat-Graphics 2.6 statistical computer program.

### Results

*Toxicity*

Patient characteristics are presented in Table I. All patients had resistant or relapsed disease, and there were no curative options left. Serum transferrin and ferritin levels were $1.83 \pm 0.19$ g l$^{-1}$ (range 1.59–2.04) and $5269 \pm 3783$ $\mu$g l$^{-1}$ (range 2060–12 444) respectively.

In total, 12 doses of $^{67}$Ga were delivered i.v. Doses ranged from 36–105 mCi. None of the patients was admitted for more than 24 h. No acute toxicity was observed. Two patients noted a slight fruity flavour. One patient noted increased bleeding tendency and muscle pains in the 4 days following 60 mCi $^{67}$Ga. However, no objective increase in bleeding tendency was observed and muscle pains did not occur after a second administration of 60 mCi $^{67}$Ga in the same patient.

No change in kidney or liver function was observed. Levels of lactate dehydrogenase (LDH) varied with disease activity, and were not correlated with $^{67}$Ga administration. Haematological effects were restricted to those on blast counts.

*Pharmacokinetics*

Table II shows the pharmacokinetics after 10 min. Most patients had whole blood levels between 1.0 and 1.5 $\mu$Ci

**Table I** Patient characteristics including age, sex, transferrin receptor density (CD71), previous therapy and disease status

| *Patient no.* | *Age* | *M/F* | *Diagnosis* | *CD71 (%)* | *Previous treatment* | *Status* |
|---|---|---|---|---|---|---|
| 1 | 27 | F | AML, M2 | | a,b,c,ABMT,d | Second relapse |
| 2 | 45 | F | AML, M1 | 23 | a,b,c,ABMT | Second relapse |
| 3 | 62 | F | AML, M1 | 18 | a(2 ×),c(2 ×),b | Resistant relapse |
| 4 | 58 | F | AML, M4 | 43 | a,b,c, + cyclosporin A | Resistant disease |
| 5 | 60 | F | AML, M5b | 79 | a(2 ×),c | Resistant relapse |
| 6 | 22 | M | ALL | 3 | e,b,d | Resistant disease |
| 7 | 32 | F | ALL | 68 | e,f,g,h | Second relapse[a] |
| 8 | 50 | M | ALL | 28 | e,f,ABMT | First relapse |

Treatments: a, daunorubicin/ARA-C; b, amsacrine/ARA-C; c, mitoxantrone/VP16 (etoposide); d, VP16 (3000 mg m$^{-2}$) + melphalan (100 mg m$^{-2}$); e, daunorubicin/vincristine/asparaiginase/prednisone; f, 6-mercaptopurine/methotrexate/ARA-C/ cyclophosphamide; g, vincristine/doxorubicin/dexamethasone; h, ARA-C/VP16. ABMT, autologous bone marrow transplantation busulphan 16 mg kg$^{-1}$ + cyclophosphamide. [a]Systemic + leptomeningeal relapse.

**Table II** Pharmacokinetics

| Patient no. | $^{67}Ga$ dose (mCi) | $^{67}Ga$ dose (mCi $m^{-2}$) | 10 min p.i. (μCi $ml^{-1}$) | 60 min p.i. (μCi $ml^{-1}$) | 24 h p.i. (μCi $ml^{-1}$) | Urine 24 h (mCi) | Urine 24 h dose (%) |
|---|---|---|---|---|---|---|---|
| 1 | 36.0 | 20 | 0.82 | 0.31 | 0.09 | NA | |
| | 62.0 | 34 | 0.45 | 0.23 | 0.09 | NA | |
| 2 | 59.6 | 31 | 1.19 | 0.58 | 0.13 | 16.3 | 27 |
| 3 | 54.9 | 34 | 1.00 | 0.45 | 0.17 | 20.7 | 38 |
| | 77.0 | 48 | 1.25 | 0.55 | 0.29 | 35.9 | 46.5 |
| 4 | 82.0 | 48 | 1.50 | 0.76 | 0.27 | 32.9 | 40 |
| 5 | 78.2 | 45 | 4.47 | 1.42 | 0.27 | 14.0 | 18 |
| | 62.6[a] | 36 | NA | NA | NA | 16.4 | 26 |
| | 83.9 | 48 | 1.86 | 0.92 | NA | 16.3 | 19.5 |
| 6 | 82.8 | 44 | 1.68 | 0.68 | 0.24 | 42.7 | 51.5 |
| 7 | 53.2 | 27 | 4.48 | 3.00 | 0.6 | NA | |
| 8 | 105.0 | 47 | 0.80 | 0.44 | 0.06 | 31.0 | 29.5 |
| | | | | | Median | 20.7 | 29.5 |
| | | | | | s.d. | 10.6 | 11.7 |

Pharmacokinetic data, including total administered dose of gallium-67 citrate (mCi), dose per square metre body surface (mCi $m^{-2}$), whole-blood $^{67}Ga$ radioactivity 10 min, 60 min and 24 h post injection (μCi $ml^{-1}$), total $^{67}Ga$ urine excretion in the first 24 h post injection (mCi) and urine excretion as percentage of the injected dose [dose (%)]. All radioactivity values are corrected for physical decay (at $t = 0$). Median value and s.d. are given for the urinary excretion. [a]$^{67}Ga$ administered as 1 h infusion. NA, not available.

$ml^{-1}$, with two patients (5 and 6) reaching higher levels of 4.5 μCi $ml^{-1}$. One h post injection (p.i.) blood levels were approximately halved, compared with 10 min p.i. The 24 h blood levels were approximately 30% of the 1 h blood levels.

One patient (patient 8) was more extensively monitored for plasma levels (Figure 1). A steep decrease in plasma radioactivity level was noted in the first minutes after administration. Pharmacokinetic data calculated in this patient are given in the legend of Figure 1.

Of the 12 administrations of $^{67}Ga$, urine excretion was measured on nine occasions. The median urine excretion of $^{67}Ga$ in the first 24 h p.i. was 29.5% (range 18–51%) of the administered dose. In three patients the first 0–6 h urine portion was collected separately. These collections contained 22.1 mCi (27% of injected dose) in patient 4, 32.8 mCi (39.6% of injected dose) in patient 6 and 17.5 mCi (16.6% of injected dose) in patient 8.

*Cellular uptake and dosimetry*

Cellular uptake values of 11 $^{67}Ga$ administrations were 1.57 ± 2.67% (range 0.07–9.00%), 1.57 ± 2.71% (range 0.05–8.70%) and 1.86 ± 2.00% (range 0.05–5.18%), 10 min, 60 min and 24 h after injection respectively (median value ± s.d.).

Cellular $^{67}Ga$ content values of 11 $^{67}Ga$ administrations were 1.73 ± 19.40 (range 0.08–66.22) $10^{-3}$ pCi per cell, 0.89 ± 4.97 (range 0.04–17.15) $10^{-3}$ pCi $cell^{-1}$ and 0.27 ± 1.25 (range 0.02–3.41) $10^{-3}$ pCi per cell, 10 min, 60 min and 24 h after injection respectively (median value ± s.d.).

The whole-body residence time was estimated to be 90.89 ± 12.40 h compared with 88.56 h calculated according to ICRP-53. We calculated a red marrow residence time of 0.41 ± 0.16 h, which differed considerably from the comparable ICRP-53 value of 4.78 h.

The whole body dose was estimated to be 0.25 ± 0.03 cGy $mCi^{-1}$, which compares well with MIRD/ICRP-53 calculations of 0.24 cGy $mCi^{-1}$. The red marrow dose depended on whether a homogeneous distribution was expected; approach A, 0.18 ± 0.02 cGy $mCi^{-1}$ or 50% accumulation of the total activity in the skeleton was assumed; approach B, 0.97 ± 0.12 cGy $mCi^{-1}$. The comparable MIRD/ICRP-53 value for the red marrow dose was 0.70 cGy $mCi^{-1}$.

*Scintigraphy*

Figure 2a shows a 5 mCi diagnostic $^{67}Ga$ scan without abnormalities; Figure 2b shows a $^{67}Ga$ scan in a non-Hodgkin's lymphoma patient after high-dose gallium-67 citrate administration; and Figure 2c shows a similar scan in patient 8. The figure shows a representative image of a scintigraphy study in a leukaemia patient 24 h after a therapeutic dose of 105 mCi of $^{67}Ga$ (patient 8). The marked increase of $^{67}Ga$ accumulation in the skeleton is notable. Decreased activity is seen in the liver, and there is almost complete absence of bowel excretion.

**Figure 1** The curve shows the course of plasma $^{67}Ga$ radioactivity values (μCi $ml^{-1}$, corrected for physical decay) in time after intravenous injection (h) of patient 8. Area under the curve, 11.46 μCi $l^{-1}$ $h^{-1}$; total clearance, 145 ml $min^{-1}$; mean residence time, 18 h; volume of distribution, 365 l; terminal half-life, 40.3 h.

**Figure 2** Whole body $^{67}Ga$ scintigraphs of diagnostic scan with a normal distribution 72 h after administration of 5 mCi $^{67}Ga$ (**a**), scintigraphs 24 h after therapeutic administrations of 100 mCi $^{67}Ga$ in a patient with non-Hodgkin's lymphoma (**b**) and patient 8 with acute myeloid leukaemia (**c**). ADAC, Genesys dual head system.

*Response*

Objective judgement of response was hindered by the concomitant anti-leukaemic medication (hydroxyurea or corticosteroid). In Figure 3 white blood cell counts (WBC) in all patients are presented in relation to the medication. AML patients 3 and 5 showed a reduction in WBC following administration of $^{67}$Ga. Normal WBC values were only reached in patient 5, although the white blood cell differentiation still showed the presence of 52% blast cells. Furthermore, notable in this patient was the normalisation of the serum LDH from 1119 U $l^{-1}$ (N = 250 U $l^{-1}$) to 155 U $l^{-1}$. The response, however, was short-lived as the WBC increased 15 days after the second administration.

Of the ALL patients, patient 6 had a definite response with WBC decreasing $<0.1 \times 10^9$ $l^{-1}$, 20 days p.i., normalisation of the enlarged spleen, which ranged 7 cm beneath the left costal margin and disappearance of skin lesions. Patient 8 had an initial rise in WBC up to over $200 \times 10^9$ $l^{-1}$ in 4 days. Dexamethasone was started followed by a steep decrease in WBC. However, it is unlikely that dexamethasone caused the ensuing leucopenia, which was probably an effect of the $^{67}$Ga administration.

**Figure 3** Course of white blood cell counts in all patients during a varying observation period. The administration of high dose gallium-67 citrate is indicated. Concomitant medication and its dose is given. In patient 5 the platelet counts are indicated as well (dotted line).

### Discussion

In the present study eight patients with relapsed and/or resistant acute leukaemia received in total 12 doses of high-dose $^{67}$Ga. No acute side-effects were observed nor any extra-haematological effect. Myelotoxicity is considered to be the major and dose-limiting toxicity in most radionuclide therapies in lymphoma patients (Kaminski *et al.*, 1993; Press *et al.*, 1993). In our study myelotoxicity was apparent by the (transient) effect on blast cells but not in changes in erythrocyte and platelet requirements.

Whole blood levels of radioactivity were unexpectedly low, with values in six patients between 0.80 and 1.86 $\mu$Ci ml$^{-1}$ 10 min after administration. We expected higher levels as $^{67}$Ga is temporarily confined to the plasma compartment by prompt binding to transferrin (Vallabhajosula *et al.*, 1980). For instance, in patient 8, with an estimated blood volume of 7.5 l and who received 105 mCi, one would have expected an initial blood level of 14 $\mu$Ci ml$^{-1}$ instead of the 0.80 $\mu$Ci ml$^{-1}$ we found. Low plasma $^{67}$Ga levels are described following chemotherapy (Shephton and Martin, 1980) but this was not the case in patient 8. The observed steep initial decrease in plasma or blood $^{67}$Ga levels seem to disagree with pharmacokinetic data of $^{67}$Ga that describes a short-lived and a long-lived component with a biological half-life of approximately 30 h and 613 ± 83 h respectively (Cloutier *et al.*, 1988). The urine excretion was unexpectedly high with a median value of 29.5% (range 18–51.5%) of the injected dose excreted in the first 24 h. The urinary excretion seems larger than the 26% of the injected dose of $^{67}$Ga in the first 7 days after injection that Nelson *et al.* (1972) observed in 23 patients.

Pharmacokinetic data of non-radioactive gallium nitrate report 24 h urinary excretion of 15–72% after a bolus injection (Hall *et al.*, 1979; Kelsen *et al.*, 1980). No difference in urinary excretion between bolus injection or 1 h infusion was observed in patient 5, who served as her own control. The observed pharmacokinetic differences could be related to the concomitant medication or differences in patient group. The iron status of our patients, who received frequent blood transfusions, could have been of influence. Another possibility is that our low-citrate formula of $^{67}$Ga influenced our data, as gallium-67 citrate can form multinucleate polymeric forms, gallium hydroxides or bind to serum proteins other than transferrin (Larson *et al.*, 1978). We tried to measure the portion of $^{67}$Ga-Trf in the plasma samples by high-performance liquid chromatography (HPLC), but could not validate sufficiently the stability of the $^{67}$Ga–Trf binding during the procedure, as free gallium-67 citrate complexes with the silica of the column.

The uptake of $^{67}$Ga in the blast cells was approximately 40 times less than in our *in vitro* experiments (Jonkhoff *et al.*, 1993, 1995; Leeuwen-Stok *et al.*, 1993). This rather low cellular $^{67}$Ga uptake might be explained by insufficient Trf-receptor expression on blast cells, the relatively high Trf concentration in the blood/bone marrow compartment, which inhibits the uptake of $^{67}$Ga in the cell (Leeuwen-Stok *et al.*, 1993) or the low blood levels of $^{67}$Ga. No apparent correlation was found between Trf receptor (CD71) expression, or $^{67}$Ga uptake in blast cells and *in vivo* response.

Our dosimetric calculations show similar whole body retention times between this study and ICRP-53. The seeming contradiction between a larger than expected urinary excretion and similar residence times to ICRP values can be explained by the neglect in our study of biological clearance after 24 h. The calculated activity in red marrow however is considerably lower than the red marrow activity suggested by the ICRP-53. The red marrow absorbed dose based on the ICRP-53 lies between red marrow absorbed dose calculations with assumed different skeleton activities (approach A or B). For individual patients the total red marrow dose varied between 10–17 cGy (approach A) and 52–110 cGy (approach B) depending on the chosen approach (data not shown). As we observed at least one definite clinical response, approach B seems more realistic. It is also possible that the absorbed whole body and red marrow doses are underestimated because microdosimetry was not taken into account (van Dieren, 1993). Furthermore, we cannot exclude the possibility that bone marrow blast cells had a higher uptake of $^{67}$Ga than the peripheral blasts, as the minimal $^{67}$Ga content in the cells with only a few disintegrations in a million cells is not likely to result in the observed clinical response.

The body distribution measured by scintigraphy showed an abnormal pattern, compared with high-dose $^{67}$Ga administration in lymphoma patients (Huijgens *et al.*, 1993). The skeleton was imaged more clearly and liver, spleen and bowel less intensively. We cannot exclude the possibility that the distribution in the skeleton is caused by the bone-seeking properties of $^{67}$Ga (Ando *et al.*, 1989) or uptake in bone marrow blasts. More likely, however, the distribution in the skeleton is due to additional factors such as iron overload. Engelstad *et al.* (1982) described a similar $^{67}$Ga distribution in patients with multiple red cell transfusions.

Responses are difficult to interpret with concomitant anti-leukaemic medication. Two AML patients seemed to respond with decreasing WBC after $^{67}$Ga administration (patients 3 and 5), but these responses seem to be short lived. Blast cells remained in peripheral blood smears. Of the ALL patients, one had a definite response with disappearance of skin lesions and normalisation of the enlarged spleen. Profound leucopenia (WBC $<0.1 \times 10^9$ l$^{-1}$) was encountered from 15 days p.i. onwards, until death 2 months later. Another ALL patient (patient 8) had a very steep decrease in WBC, with disappearance of blast cells, following dexamethasone medication. This decrease in WBC might be caused by the $^{67}$Ga administration, as radionuclide therapy is known to exert its effect only after several days. In total, we observed one definite response (13%) and three possible responses (38%) out of eight patients.

Our conclusion is that high-dose $^{67}$Ga therapy is well tolerated, but cellular $^{67}$Ga uptake is relatively low. Pharmacokinetic data suggest a large proportion of non-transferrin-bound $^{67}$Ga, influencing urine excretion, body distribution and possibly cellular uptake. Nevertheless, transient responses and one definite response were noted. Therefore, we feel that if cellular $^{67}$Ga uptake can be enhanced by additional measures, such as desferrioxamine or iron–dextran administration (Shani *et al.*, 1986), high-dose $^{67}$Ga therapy might be useful in leukaemia patients.

#### Acknowledgements
This study was supported by a grant from the Dutch Cancer Society (IKA 91-07).

### References

ANDO A, ANDO I, HIRAKI T AND HISADA K. (1989). Relation between the location of elements in the periodic table and various organ-uptake rates. *Nucl. Med. Biol.*, **16**, 57–80.

ANGHIERI LJ AND HEIDBREDER M. (1977). On the mechanism of accumulation of $^{67}$Ga by tumors. *Oncology*, **34**, 74–77.

APPELBAUM FR, MATTHEWS DC, EARY J, FISHER L, PRESS O, MARTIN P, DURACK L, BADGER C AND BERNSTEIN I. (1990). Use of radioiodinated anti-CD33 antibody to augment marrow irradiation prior to marrow transplantation for AML. *Proc. Am. Soc. Hematol.*, **76**, 526a.

CHITAMBAR CR AND SELIGMAN PA. (1986). Effects of different transferrin forms on transferrin receptor expression, iron uptake, and cellular proliferation of human leukaemic HL60 cells. *J. Clin. Invest.*, **78**, 1538–1546.

CLOUTIER RJ, WATSON EE, HAYES RL, NELSON B AND SMITH EM. (1988). Report No. 2: Gallium-66-, Gallium-67-, Gallium-68-, and Gallium-72-citrate. In: *MIRD Primer, for Absorbed Dose Calculations*, Loevinger R, Budinger ThF and Watson EE (eds), pp. 40–42, Society for Nuclear Medicine: New York.

COURSEY BM, CALHOUN JM, CESSNA J, GOLAS DB, GRAY DH, HOPPES DD, SCHIMA FJ AND UNTERWEGER MP. (1991). National standards for diagnostic and therapeutic nuclides. In: *Fifth International Radiopharmaceutical Dosimetry Symposium.* Proceedings of a conference held at Oak Ridge (TN): 7–10 May, 1991. pp. 152–143.

ENGELSTAD B, LUK SS AND HATTNER RS. (1982). Altered $^{67}$Ga distribution in patients with multiple red blood cell transfusions. *AJR*, **139**, 755–759.

HALL SW, YEUNG K, BENJAMIN RS, STEWART D, VALDIVIESO M, BEDIKIAN AY AND LOO TL. (1979). Kinetics of Gallium nitrate, a new anticancer agent. *Clin. Pharmacol. Ther.*, **25**, 82–87.

HOFER KG, HARRIS CR AND SMITH JM. (1975). Radiotoxicity of intracellular 67-Ga, 125-I and 3-H nuclear versus cytoplasmic radiation effects in murine L1210 leukaemia. *Int. J. Radiat. Biol.*, **28**, 225–241.

HUIJGENS PC, JONKHOFF AR, HOEKSTRA OS, OSSENKOPPELE GJ AND TEULE GJJ. (1993). Therapeutic potential of intravenous 67-Gallium in non-Hodgkin's lymphoma. *Eur. J. Haematol.*, **51**, 206–208.

INTERNATIONAL COMMISSION ON RADIATION PROTECTION (ICRP). (1987). Radiation dose to patients from radiopharmaceuticals. ICRP publication no. 53. *Annu. ICRP*, **18**, 1–4.

JONKHOFF AR, HUIJGENS, PC, VERSTEEGH RT, VAN DIEREN EB, OSSENKOPPELE GJ, MARTENS HJM AND TEULE, GJJ. (1993). Gallium-67 radiotoxicity in human U937 lymphoma cells. *Br. J. Cancer*, **67**, 693–700.

JONKHOFF AR, VAN DIEREN EB, HUIJGENS PC, VERSTEEGH RT, DRÄGER AM, VD LOOSDRECHT AA AND TEULE GJJ. (1994). Biological effectiveness of 67-Gallium decay in HL60 cells compared with external low dose rate gamma irradiation: effects on proliferation, G2-arrest and clonogenic capacity. *Int. J. Radiat. Oncol. Biol. Phys.*, **30**, 117–124.

JONKHOFF AR, HUIJGENS PC, VERSTEEGH RT, VAN LINGEN A, OSSENKOPPELE GJ, DRÄGER AM AND TEULE GJJ. (1995). Radiotoxicity of 67-Gallium on myeloid leukaemic blasts. *Leukaemia Res.*, **19**, 169–174.

KAMINSKI MS, ZASADNY KR, FRANCIS IR, MILIK AW, ROSS CHW, MOON SD, CRAWFORD SM, BURGESS JM, PETRY NA, BUTCHKO GM, GLENN SD AND WAHL RL. (1993). Radioimmunotherapy of B-cell lymphoma with [$^{131}$I]anti-B1 (anti-CD20) antibody. *N. Engl. J. Med.*, **329**, 459–465.

KELSEN DP, ALCOCK N, YEH S, BROWN J AND YOUNG CH. (1980). Pharmacokinetics of Gallium nitrate in man. *Cancer*, **46**, 2009–2013.

LARSON SM, ALLEN DR, RASEY JS AND GRUNBAUM Z. (1978). Kinetics of binding of carrier-free Ga-67 to transferrin. *J. Nucl. Med.*, **19**, 1245–1249.

LEEUWEN-STOCK, VAN AE, DRÄGER AM, SCHUURHUIS GJ, PLATIER AWJ, TEULE GJJ AND HUIJGENS PC. (1993). Gallium-67 in the human lymphoid cell line U-715: uptake, cytotoxicity and intracellular localization. *Int. J. Radiat. Biol.*, **64**, 749–759.

MAKRIGIORGOS G, ADELSTEIN SJ AND KASSIS AI. (1990). Auger electron emitters: insight gained from in vitro experiments. *Radiat. Environ. Biophys.*, **29**, 75–91.

NATIONAL COUNCIL ON RADIATION PROTECTION AND MEASUREMENTS. (1970). *Precautions in the Management of Patients who have Received Therapeutic Amounts of Radionuclides*, NCRP report no. 37. National Council on Radiation Protection and Measurements: Washington, DC.

NELSON B, HAYES RL, EDWARDS CL, KNISELEY RM AND ANDREWS GA. (1972). Distribution of gallium in human tissues, after intravenous administration. *J. Nucl. Med.*, **13**, 92–100.

PLAIZIER MABD, ROOS JC, TEULE GJJ, VAN DIEREN EB, DEN HOLLANDER W, HAISMA HJ, DEJAGER RL AND VAN LINGEN A. (1994*a*). Comparison of non-invasive approaches to red marrow dosimetry for radiolabelled monoclonal antibodies. *Eur. J. Nucl. Med.*, **21**, 216–222.

PLAIZIER MABD, ROOS JC, VAN DIEREN EB AND TEULE GJJ. (1994*b*). Radionuclide therapy and the influence of activity distribution on bone marrow dose (abstract). *Eur. J. Nucl. Med.*, **21**, 984.

PRESS OW, EARY JF, APPELBAUM FR, MARTIN PJ, BADGER CHC, NELP WB, GLENN S, BUTCHKO G, FISHER D, PORTER B, MATTHEWS DC, FISHER LD AND BERNSTEIN ID. (1993). Radiolabeled-antibody therapy of B-cell lymphoma with autologous bone marrow support. *N. Engl. J. Med.*, **329**, 1219–1224.

RAO DV AND HOWELL RW. (1993). Time-dose-fractionation in radioimmunotherapy: implications for selecting radionuclides. *J. Nucl. Med.*, **34**, 1801–1810.

ROHATINER AZS AND LISTER TA (eds). (1990). The treatment of acute myelogenous leukaemia. In: *Leukaemia*, 5th edn. p. 485. WB Saunders: Philadelphia.

ROSEN ST AND KUZEL TM. (eds). (1993). *Immunoconjugate therapy of hematological malignancies.* Kluwer: Dordrecht, The Netherlands.

SCHWARTZ MA, LOVETT DR, REDNER A, FINN RD, GRAHAM MC, DIVGI CR, DANTIS L, GEE TS, ANDREEFF M, OLD LJ, LARSON SM AND SCHEINBERG DA. (1993). Dose-escalation trial of M195 labelled with Iodine 131 for cytoreduction and marrow ablation in relapsed or refractory myeloid leukaemia. *J. Clin. Oncol.*, **11**, 294–302.

SEPHTON R AND MARTIN JJ. (1980). Modification of distribution of gallium 67 in man by administration of iron. *Br. J. Radiol.*, **53**, 572–575.

SHANI J. (1986). Drugs that alter biodistribution and kinetics of radiopharmaceuticals. In: *Fourth International Radiopharmaceutical Dosimetry Symposium.* Schlafke-Stelson AT and Watson EE (eds). Proceedings of a conference held at Oak Ridge (TN); 5–8 November 1985. pp. 299–300.

SIEGEL JA, WESSELS BW, WATSON EE, STABIN MG, VRIESENDORP HM, BRADLEY EW, BADGER CC, BRILL AB, KWOK CS, STICKNEY DR, ECKERMAN KF, FISHER DR, BUCHSBAUM DJ AND ORDER SE. (1990). Bone marrow dosimetry and toxicity for radioimmunotherapy. *Antibody Immunoconj. Radiopharmacol.*, **3**, 213–233.

TANSWELL P AND KOUP J. (1993). Topfit: a PC-based pharmacokinetic/pharmacodynamic data analysis program. *Int. J. Clin. Pharmacol. Ther. Toxicol.*, **31**, 514–520.

VALLABHAJOSULA SR, HARWIG JF, SIEMSEN JK AND WOLF W. (1980). Radiogallium localization in tumors: blood binding and transport and the role of transferrin. *J. Nucl. Med.*, **21**, 650–656.

VAN DIEREN EB. (1993). *Dosimetry of Radionuclides Applicable for Cancer Therapy, Based on Distance Histogram Techniques* (Thesis). Free University, Amsterdam, The Netherlands.

WATSON EE AND STABIN M. (1984). Basic alternatives software package for internal radiation dose calculations. In: *Computer Applications in Health Physics*, Kathren RL, Higby DP and McKinney MA (eds) pp. 4.49–4.58, Proceedings of the 17th Midyear Topical Symposium of the Health Physics Society; Richland, Washington, Columbia Chapter, HPS.